# Catastrophic Prosthetic Valve Endocarditis Caused by Rare Black Fungi

**DOI:** 10.1155/2018/1758539

**Published:** 2018-09-30

**Authors:** Khurram Butt, Ranjeet Kumar, Jason D'Souza, Joseph Limback, Rajesh Shah, Jeremy Burt

**Affiliations:** Florida Hospital, Orlando, USA

## Abstract

Fungal infection of prosthetic heart valves is rare and can lead to severe complications including death. Dematiaceous mold, also known as “black fungi,” are an extremely rare cause of endocarditis that usually affect immunocompromised hosts. The infection is usually chronic and can lead to heart failure and embolic complications. These fungi have limited antifungal treatment modalities. We present a rare case of prosthetic aortic valve, root, and graft infection in an immunocompetent host that revealed itself through renal, mesenteric, and cerebral embolic phenomenon. The patient underwent removal and replacement of the aortic graft followed by small bowel resection for mesenteric infarction. Patient had a successful postoperative course and underwent a long-term antifungal treatment with amphotericin B and voriconazole.

## 1. Introduction

Fungal infection of native and prosthetic cardiac valves in an immunocompetent patient is a rare occurrence and is a rather dreaded disease. Its estimated incidence can be as high as 2.9% [1] and mortality rate ranges from 23 to 46% [2–5]. The most common fungal agents affecting native heart valves are Candida, Aspergillus, and Histoplasma with Candida species being the most common amongst them [6, 7]. We hereby present a rare case of fungal prosthetic valve endocarditis (PVE) and ascending aortic root graft infection in an immunocompetent host caused by dematiaceous mold.

## 2. Case Presentation

**Figure 1:**
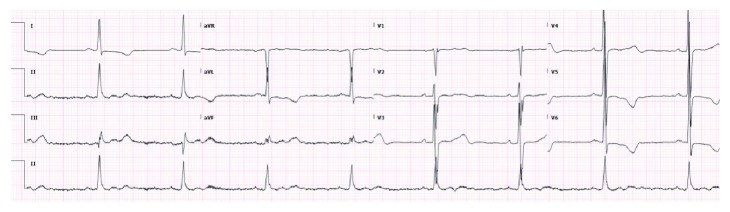
Electrocardiogram showing sinus bradycardia and left ventricular hypertrophy.

**Figure 2:**
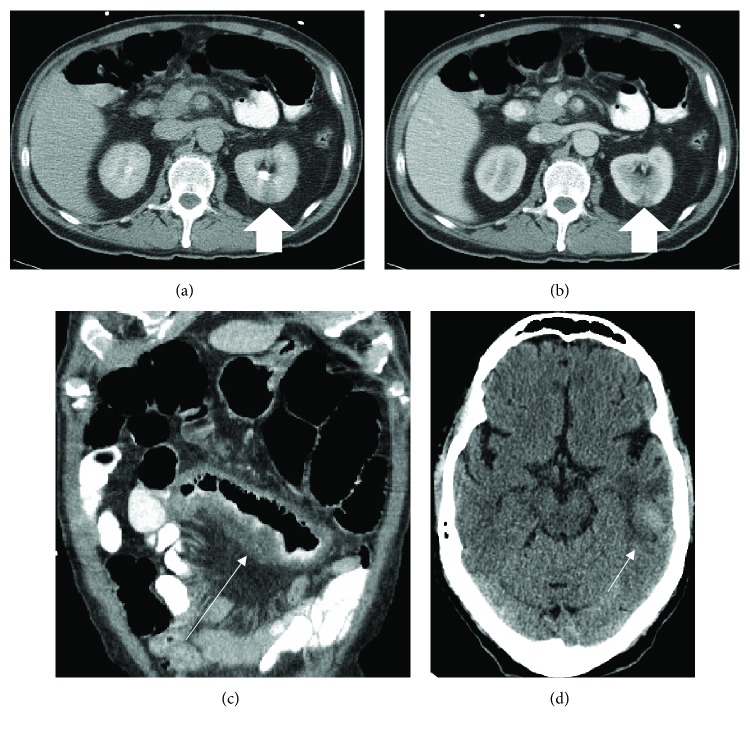
(a, b) Axial contrast-enhanced abdominal CT demonstrates a segmental infarct in the posterior aspect of the left kidney (short thick arrow). (c) Coronal reformatted CT showing infarcted small bowel (long thin arrow). (d) Noncontrast head CT showing a subacute hemorrhagic infarct in the left temporal lobe (short thin arrow).

**Figure 3:**
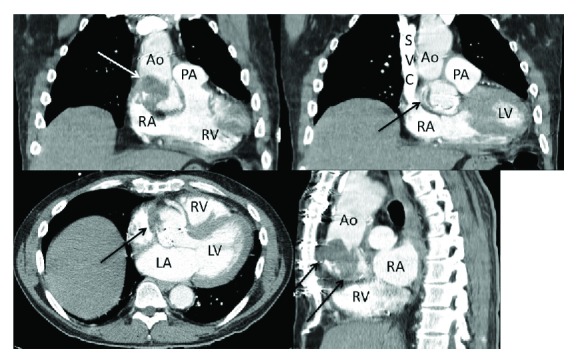
Axial, sagittal, and coronal contrast-enhanced CT images of the chest showing a fungal ball in the aortic root adherent to the prosthetic valve (arrows). Ao = aorta; PA = main pulmonary artery; RA = right atrium; LA = left atrium; RV = right ventricle; LV = left ventricle; SVC = superior vena cava.

A 68-year-old male with past medical history of coronary artery disease status post coronary artery bypass graft (CABG), aortic valve replacement along with replacement of the root of the ascending aorta 10 months prior to presentation and recent hemorrhagic cerebrovascular accident (CVA), came to the hospital with complains of acute onset of severe abdominal pain and melena for 1 day. He also attested to chronic abdominal pain and a 30-pound weight loss over the last 8 months prior to these acute symptoms. His physical exam on presentation was positive for severe bilateral lower abdominal tenderness. Apart from a hemoglobin of 10 mg/dl and a positive stool occult blood test, the rest of his basic lab work up was unremarkable (white blood cell/platelet count, comprehensive metabolic panel, and PT/INR included). Hepatitis B, hepatitis C, and human immunodeficiency virus (HIV) testing were negative. The electrocardiogram (EKG) showed sinus rhythm and left ventricular hypertrophy (Figure 1). An emergent computerized tomography (CT) scan of the abdomen revealed features suggestive of an embolic infarct in the left kidney (Figures 2(a) and 2(b)) and within the mid-one-third of the superior mesenteric artery causing luminal narrowing and also suspected to be extending to the takeoff of small bowel branches. Segmental mural thickening of at least one small bowel loop was noted which strongly favored acute bowel ischemia as a cause of his abdominal pain (Figure 2(c)). Incidental findings on CT of the abdomen were also strongly suspicious for large eccentric thrombus in the ascending aortic graft and the aortic root which were further investigated and confirmed with a CT scan of the chest (Figure 3). Cardiology and cardiothoracic surgery were consulted. A CT scan of the head was performed to assess the recent CVA and showed a subacute hemorrhage along the left-sided temporal parenchyma (Figure 2(d)). CT head imaging was obtained from the facility where the patient presented 3 months prior for cerebral hemorrhage and in comparison, to the most recent CT scan of the head, the hemorrhage appeared stable. The hemorrhage was suspected to be secondary to thromboembolism. After a review of the risks and benefits of anticoagulation to prevent extension of this suspected thrombus, heparin was initiated. An echocardiogram revealed dilatation of the ascending aorta and mild paravalvular leak around the bioprosthetic aortic valve. Gastroenterology was consulted and an emergent esophagogastroduodenoscopy was performed which was negative for any causes of upper gastrointestinal bleed. A hypercoagulable workup was performed which did not reveal any apparent cause of a prothrombotic state. Anticoagulation was held and subsequently, the patient underwent a redo sternotomy under cardiopulmonary bypass with extensive lysis of adhesions, removal of the thrombosed aortic valve and graft, ascending and proximal aortic arch replacement utilizing a 30 mm Dacron graft, and aortic valve replacement with a 25 mm Edwards Magna Ease bovine pericardial valve. The patient also underwent an explorative laparotomy as a part of a staged procedure to address the ischemic bowel caused by the presumed septic emboli. Intraoperatively, the patient was found to have a portion of small bowel that had become necrotic. The necrotic bowel was excised and an end to end anastomosis was performed.

**Figure 4:**
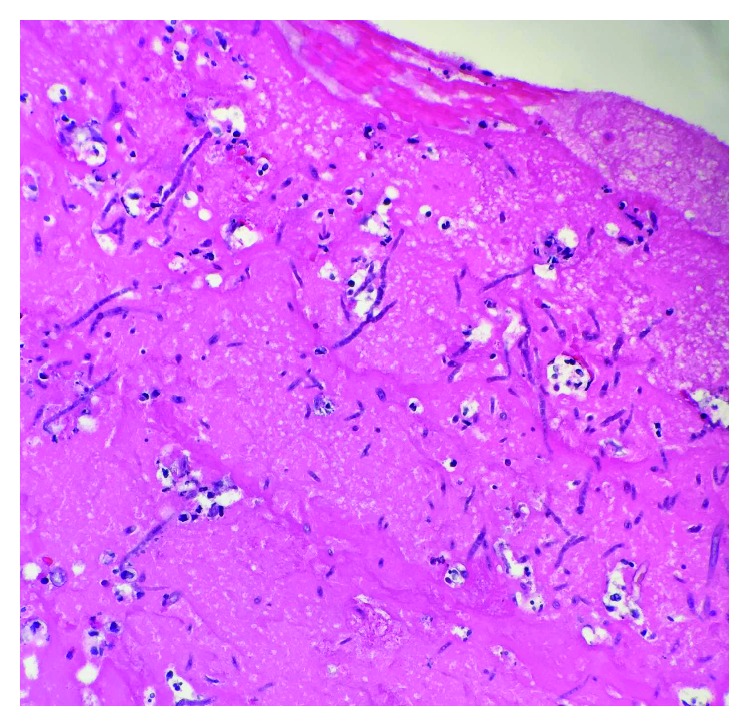
Histopathological sample of the aortic graft shows chronic inflammation and fungus with acute angle branching hyphae.

The aortic graft and thrombus were sent for culture and pathology. Histopathological examination of the aortic graft and cusps of the aortic valve revealed chronic inflammation and was also notable for abundant acute angle branching septate fungal hyphae (Figure 4). The patient was immediately started on amphotericin B and voriconazole pending finalization of cultures and sensitivities. On postoperative day four, three culture reports from the graft came back positive for dematiaceous mold, suggestive of Bipolaris species. The minimum inhibitory concentration (MIC) was 0.25 ug/dl for voriconazole and 0.03 ug/ml for amphotericin B. A decision was made to continue the same antifungal regimen on the basis of sensitivities and further speciation was not performed. Patient had a good postoperative course and was later discharged on amphotericin B and voriconazole for at least 1 month with continued follow-up with an infectious disease specialist.

## 3. Discussion

Dematiaceous mold, also known as “black fungi” owing to the presence of melanin like pigments, are a heterogeneous group of filamentous molds. These saprophytic and plant pathogenic fungi are now increasingly being recognized as causes of human infection, particularly in immunocompromised persons, such as diabetics, transplant recipients, and patients with human immunodeficiency virus (HIV) infection [8]. Dematiaceous molds are often found in soil and thus have a worldwide distribution. These molds cause a heterogeneous group of infections referred to as phaeohyphomycosis which include local infections, pneumonia, brain abscesses, and disseminated infections. Phaeohyphomycosis is characterized by the presence of black-pigmented hyphae or pseudohyphae.

**Figure 5:**
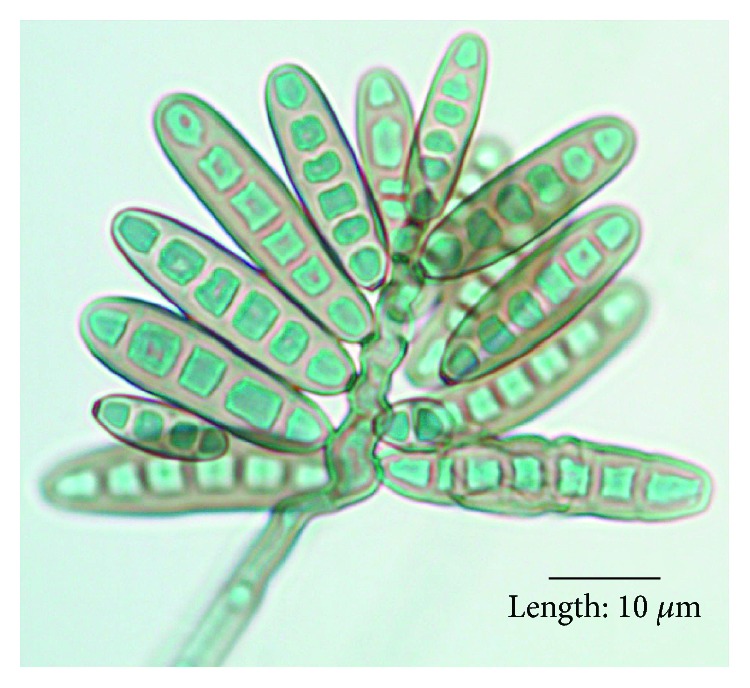
Image demonstrating conidia of Bipolaris hawaiiensis [16].

Bipolaris genus belongs to the group of dematiaceous molds and has more than 100 species. These organisms are mostly pathogens of plants; however, a few saprobic species are potentially able to infect humans and animals. Morphologically, Bipolaris species are differentiated by their rapidly growing dark colonies, large conidia with transverse distosepta (Figure 5), and bipolar method of germination. Bipolaris species are an extremely rare cause of endocarditis and aortic root infection, especially in an immunocompetent host. These infections evolve chronically and predispose to thromboembolic phenomenon which can be the primary manifestation of infection as evidenced by our case.

Vallabhaneni et al. [9] identified 21 cases of surgical site infection caused by Bipolaris species in over ten hospitals located in Texas, Arkansas, and Florida out of which 6 cases involved valve replacement. Pauzner et al. [10] reported a case of homograft aortic valve infected with Bipolaris spicifera now termed as “Curuvularia spicifera” which was treated with surgical valve replacement and antifungal medications. The patient had a good long-term outcome. Ogden et al. [11] also reported fungal endarteritis of the ascending aorta by Bipolaris species following replacement of the aortic valve with a porcine valve which ultimately resulted in the patient's death. Our case represents a rare success story with a successful treatment outcome in endocarditis/endarteritis caused by dematiaceous mold in an immunocompetent patient managed aggressively with surgical and antifungal treatments.

The European Society of Clinical Microbiology and Infectious Diseases (ESCMID) and European Confederation of Medical Mycology (ECMM) guidelines recommend using voriconazole, posaconazole, itraconazole, or in some cases amphotericin B to target infections caused by dematiaceous molds [12]. However, the current antifungal treatment modalities for patients infected with dematiaceous mold are limited, and the outcome of antifungal treatment appears dismal. In one survey, it was shown that only 23% of the patients treated with amphotericin B survived [13]. Itraconazole has been shown to be active against many species of the dematiaceous molds and there are case reports of successful treatment of some patients with systemic forms of phaeohyphomycosis, such as arthritis and osteomyelitis, including those refractory to other agents [14]. Treatment with newer agents, such as voriconazole, or treatment with combinations of antifungal agents may prove to be encouraging in the management of these infections [15]. Due to limited data on sensitivities and treatment response, usually, a two or more drug combination is employed and treatment continued under supervision of an infectious disease specialist after discharge from the hospital.
